# Sustainability-oriented innovation system and economic stability of the innovative countries

**DOI:** 10.3389/fpubh.2023.1138034

**Published:** 2023-06-22

**Authors:** Faiza Manzoor, Longbao Wei, Qazi Abdul Subhan, Mahwish Siraj

**Affiliations:** ^1^Department of Agricultural Economics and Management, School of Public Affairs, Zhejiang University, Hangzhou, China; ^2^Bahria Business School, Bahria University, Islamabad, Pakistan; ^3^Allama Iqbal Open University, Islamabad, Pakistan

**Keywords:** sustainability oriented innovation system, economic stability, innovative countries, innovation input index, innovation output index

## Abstract

Novelcoronavirus-19 has created a challenging situation for developed as well as developing countries to sustain economic stability. There are a lot of controversies for policymakers to formulate an effective policy for reviving economic stability and minimizing the economic effects of this pandemic. The present study focuses on the internal mechanism of the Sustainability Oriented Innovation System and its subsequent effects on economic stability in most innovative economies. For empirical analysis of the most innovative countries (12 countries) high-income, middle-income, low-income, and lower-middle-income countries are selected. The Sustainability Oriented Innovation System is represented through the innovation input index and innovation output index. Economic stability is measured through the GDP growth rate of respective countries. A set of panel data was developed for the period of 11 years and Fixed Effect Methods were used to ascertain the empirical findings. The outcomes indicate that innovation is the main force of economic stability. The study’s results are important to policymakers to promote, stimulate and support economic stability through their strategies. Future studies may focus on the effects of the Sustainability Oriented Innovation System on economic stability in regional blocks like the EU, ASEAN, and G-20 countries.

## 1. Introduction

The Novelcoronavirus-19 has distressed the global economy and obligated policymakers to restate their preferences and policy measures for sustainable economic development. It has pushed the world economies into deep and serious recessions (1). COVID-19 has drastic and acute effects on the economies of developing countries, which were already in serious economic, social, and political crunches. According to World Economic Outlook (2), the global economic growth rate is expected to decline by 4.9 percent in the fiscal year 2020–21. The World Economic Outlook has projected a very high degree of uncertainty in the aggregate demand of the global economy. Due to this pandemic, economic activities in developing as well as developed economies have adverse effects on key economic parameters like employment, investment, and growth in the industrial and agriculture sectors (3). Before this pandemic, United Nations has announced the Agenda 2030 in 2015 to transform the global economy for sustainable economic development. Under this Agenda 2030, 17 sustainable development goals (SDGs) and 169 targets have been announced. These goals are integrated and are also based on Millennium Development Goals (MDGs). Each country is having its challenges and opportunities which can be addressed with political commitment, consistent policy formulation for sustainable development, and implementation of these policies in true letters and spirit (4, 5). Furthermore, Ghassim and Bogers (6) have emphasized the importance of the Sustainability Oriented Innovation System (SOIS) for achieving SDGs in developed and developing countries.

There is no perfect prediction about the longevity of this worldwide pandemic, which has been started in 2019. It has deep-rooted and prolonged adverse effects globally. Relatively, developing economies are more affected than developed economies (7, 8). The World Bank has published its flagship report about Global Economic Prospects (2020) and identified the critical situations of developing countries during the Pandemic 2019. The report further explains that global growth is expected to decline by 5.2 percent and this pandemic is considered as deepest global recession since Second World War. All economic and social indicators in the globe are showing declining trends (9, 10). The report has further highlighted multiple sectors to improve the economic situation and make the economies more resilient to cope with the situations in the future. The main areas of improvement are health sector facilities, policy formulations, and more elastic innovative systems. The report emphasized global cooperation to save vulnerable populations in developing countries and improve the country’s capacity to cope with this situation in the future (11). Similarly, this study is concerned, it focuses on the current economic situations of developed and developing countries during COVID-19. Secondly, it will discuss UN Agenda 2030 for sustainable development among member countries, and thirdly, the role of the SOIS in achieving economic stability in the most innovative economies. All these three aspects are integrated in such a way that one aspect reflects the prevailing economic and social conditions of the economies around the globe. Second is the objective that should be achieved, and all the economies unanimously agreed to achieve sustainable economic growth. The third aspect is a strategy and technique through which countries can achieve their objectives more efficiently and effectively. This study is important in a way that it covers all aspects of global as well as regional economies regarding prevailing situations. Therefore, the main aim of the present study is an attempt to investigate the impact of SOIS on economic stability in developing countries in the context of the UN Agenda 2030. This study analyzes the status of achievements against Agenda 2030 and the magnitude of devastation due to COVID-19. The global economy is consistently facing multiple challenges on economic, political, and social fronts. To cope with these challenges, there are multiple recommendations by international agencies. More specifically, the current pandemic has compelled policymakers to reframe their policies for long-term development. Implementing a SOIS is one possible solution. The present study further explains the status of developing countries, their bottlenecks, and the deliberation of a possible mechanism for the implementation of SOIS for economic stability in respective countries. This study’s findings are the first of their kind in the study region. The study’s specific research questions are as follows:

- *What is the status of the economic stability of the most innovative countries in the current pandemic situation?*
- *What are the current achievements of respective countries against the UN Agenda 2030?*
- *What are the main components of the Sustainability Oriented Innovation System?*
- *How to develop an effective mechanism in the implementation of the Sustainability Oriented Innovation System to attain the objectives of economic stability in developing countries.*

Economic stability is one of the primary goals of all countries. Economic stability can be interpreted as sustainable economic growth, price stability, and employment opportunities (12). An effective management policy can stabilize the economy. The main purpose of this study is to identify the status of economic stability in the most innovative countries. It is admitted fact that innovation is widely recognized as a key driver of economic growth, competitiveness, and social progress. High-income countries, which are generally characterized by strong research and development (R&D) capabilities, highly skilled workforces, and advanced technology infrastructure, have established robust innovation systems that facilitate the creation, diffusion, and commercialization of new knowledge and technologies. These systems typically involve a range of actors, including universities, research institutions, private firms, and government agencies, who collaborate to generate, disseminate, and apply new ideas and technologies (13). High-income countries have invested heavily in innovation over the years, through public funding of research and development, tax incentives for private R&D, and other policies. The respective governments have designed those programs which support the innovation systems. As a result, these countries have been able to generate new products, services, and business models that have helped to drive economic growth and enhance social welfare. However, innovation systems are not static and continue to evolve in response to changing economic, social, and technological conditions, which require ongoing efforts to improve them.

Similarly, innovation systems in low-income countries are often underdeveloped due to a lack of resources, infrastructure, and access to technology (14). To address these issues, governments and international organizations have implemented a variety of initiatives to foster innovation and economic growth. These initiatives include the provision of access to finance, creating incentives for research and development, and encouraging collaboration between the public and private sectors. Additionally, initiatives like Global Innovation Fund are helping to provide resources and support to innovators in low-income countries. These initiatives are helping to create an environment that encourages innovation and economic growth, and ultimately, improves the lives of people in low-income countries. This study will help assess the current challenges of the respective economies and how SOIS may foster innovation-driven economic growth in their respective countries.

The paper is organized in the following manner. The introduction section is followed by a brief review of the literature, methodology and data, results, and discussions. In the last section, the conclusion and recommendations are presented with practical implications and limitations.

## 2. Literature review

This section covers the current economic situations of developed and developing countries during COVID-19, the UN Agenda 2030 for sustainable development, and the role of the SOIS in achieving economic stability in developing economies.

### 2.1. An overview of the global economic situation during COVID-19

COVID-19 has embraced astonishing effects on the global economy and pushed the world economies into a deeper economic and social crisis for which the world was neither prepared nor imagined. This crisis has created multidimensional problems in the health sector, employment sector, poverty issues, adverse law and order issues, depravedness of necessities, a vulnerability in the societies, and problems in the nonavailability of medical treatments in the hospitals’ (7, 15). This pandemic has created deep-rooted damages to the determinants of economic growth prospects in the world economy and has ruined the standard of living and created a deep recession (16). The World Bank published a Flagship report on Global Economic Prospects in 2020. The report has comprehensively discussed the effects of COVID-19 on global and regional economies. This pandemic has resulted in a huge contraction in financial conditions worldwide. The global economic growth contraction is expected to be 5.2 percent during the financial year 2020–21. The advanced economies are expected to reduce their economic growth by 7.2 percent. Among the advanced economies, the EU has been rigorously affected and their economies are projected to contract by around 9.1 percent (17). The real GDP of High-Income Countries is projected to reduce by 6.8 percent as compared to a contraction of developing economies by 2.4 percent during FY 2020. The world trade volumes have been constricted by up to 13.4 percent which has left a majority of an unemployed and vulnerable population. The low-income countries are expected to grow by 1%. One of the drastic impacts has been observed on oil prices which have reduced by 47.9 percent due to a reduction in international oil demand (18).

All major sectors of advanced economies have been disrupted due to COVID-19. The second wave of this pandemic is on its high surge which may further aggravate the intensity of economic, social, and financial conditions of the economies (19). For instance, the US economy is expected to contract by more than 6.1 percent which has very serious repercussions on its economic and social fronts. The people face high unemployment and rising inflation. There was a massive reduction in retail sales and industrial production during COVID-19. Similarly, the economic growth in emerging economies like Malaysia, China, India, and Thailand has worsened more than expected in the first quarter of 2020 and there is a high probability that it will further decline with massive magnitude in the second quarter (2). The worst effects of this pandemic have been observed in developing economies. For the first time, all the regions projected negative economic growth in FY 2020. All the developing countries have structural differences but particularly, South Asian countries have faced longer lockdowns for more than 6 months which has created a massive decline in aggregate demand and an increase in precautionary savings. In other words, capital formation in developing countries has drastically declined and it has hit the employment sector severely. More than 6.65 million people have become unemployed only in Pakistan during the fiscal year 2020–21 (20). These adverse effects have been observed in all developing nations. For instance, the Indian economy has suffered severely due to Coronavirus-19 and its unemployment went up to 24 percent in Fiscal Year 2020. Due to strict lockdowns in major cities, all economic activities were suspended. There are three main sectors; agriculture, services, and manufacturing sectors, which were directly hit due to COVID-19 in all South Asian Economies (21). When the industrial sector remains closed then there is no question of employment and a high surge in unemployment was observed in the Indian economy during FY 2020–21. The second wave of this pandemic has knocked down the Indian economy to its record level. In April 2021, the number of COVID Cases spiked up to 350,000 per day and the death toll has surged by more than 2,700 per day. This emergency has collided with the whole infrastructure of the Indian economy. The basic reasons behind this situation are the unprecedented intensity of the pandemic, the wrong perception of the people about COVID-19, the lack of public health infrastructure, and the lack of intensive care regarding the SOPs (Standard Operating Procedures) related to COVID-19 (22). Moreover, due to COVID-19 manufacturing, services, and Micro, Small, and Medium Sized Enterprises (MSMEs) sectors have shown a drastic decline in their respective growths (23, 24). Shafi et al. (25) have investigated the effects of COVID-19 on small and medium enterprises in Pakistan. They have observed a sharp decline in sales growth, supply chain, decrease in demand, and reduction in profitability of the MSMEs (24, 26). The almost same situation has prevailed in other developing countries (27).

This pandemic has not given any relaxation to developed economies despite having good health infrastructure, capital availability, and advanced technologies. All European economies, especially Italy, France, and Germany have been seriously affected due to COVID-19. A high death toll has been observed in these countries daily (28). The doctors and other paramedical staff have sacrificed their lives during their official duties and strived their best to recover the patients. Similarly, USA’s economy has suffered a serious setback on economic and social fronts. The economic indicators have reflected negative growth in major sectors of the economy. For instance, real GDP growth in the second quarter of FY 2020 in the USA has declined up to 31.40 percent. The unemployment rate has spiked up to 14.7 percent which was not observed since the era of the Great Depression. These adverse numbers have created serious distress in the US economy on economic, social, and political fronts.

### 2.2. The UN Agenda 2030 for sustainable economic development

In the presence of global recession and slow economic activities, The UN Agenda 2030 is one of the policy guidelines for policymakers. The Agenda 2030 is presumed as a plan of action for the prosperity and nourishment of all countries without any discrimination for the achievement of sustainable development. This Agenda has 17 SDGs and 169 targets. The focus of these Agenda items is the development of human capital, poverty eradication, prosperity, and sustainability with the help of innovative and technological signs of progress, planet safety through optimal utilization of natural resources and minimizing environmental deregulations, fostering peace and harmony among the countries, and establishing global partnerships among the countries for sustainable development (29). These sustainable development goals are interlinked and accomplishing these objectives has significant importance for transforming global miseries. Especially, to minimize the economic sufferings of COVID-19, there is a dire need to focus on these Agenda items and initiate the execution of these objectives in practicality (30).

First and foremost, the area of concentration of Agenda 2030 is to keep the world free of poverty hunger, and diseases. This item mainly addresses the social infrastructure of the countries like health facilities, education provisions, and economic opportunities. There are certain prerequisites for maintaining minimum benchmarks for creating conducive environments for social facilities. For instance, the vision of government, the status of industrialization within the country, and prevailing health and education facilities. The vision of the government plays a vital role in the accomplishment of these goals (31). If the governments have no vision to develop their respective nations, as has been observed in most of the developing countries, then these objectives become just a dream and the inhabitants of these nations may suffer all sorts of hardships of poverty, hunger, and poor health infrastructure. During the current pandemic, the most affected sectors are health, services, and manufacturing in the world (3).

There is an immense need to revitalize the policies for social development. The UN Agenda 2030 report has discussed the stunning challenges for sustainable development like economic disparities among the countries, income disparities, poverty, problems of gender inequality, health disparities, frequent natural disasters, unemployment issues, terrorism, sectarian violence, natural resource depletion, and humanitarian crisis. It is a matter of fact that these challenges can never be controlled until and unless a comprehensive and cohesive policy framework has not been formulated. The policy formulation may be segmented into short-term, medium-term, and long-term perspectives. For short-term policy objectives, all the countries must concentrate on the remedial measures for this fatal pandemic that has paralyzed the global economy. For instance, the vaccination process should be faster without any further delays and discrimination. This process was accomplished in some countries like England, and Israel, and now the people of these countries are getting a sigh of relaxation but most of the European countries, South Asian, and Southeast Asian economies are still in the clutches of COVID-19. In this regard, the role of the World Health Organization (WHO) is exemplary. WHO has generated COVID-19 Solidarity Response Fund to assist the member economies in caring for the patients, frontline workers supply, and providing relevant information about medical research.

For medium-term objectives, the public sector of developing economies ought to focus on health facilities on a prior basis. These economies have focused on increasing health expenditures up to a minimum of 5 percent of their respective Gross Domestic Product (GDP) to face such kind of severe pandemics in the future. To attain SDGs, more particularly, SDG-16 and SDG-17 which are concerned with peaceful and inclusive societies and to strengthen the means of implementation to promote global partnership for sustainable development among the member countries, The United Nations should play an exemplary role to resolve the deadlocks between respective countries like the matter of Palestine, dispute of Kashmir between India and Pakistan and other prominent issues among the countries under the charter of UN. When these countries will become out of these issues then they may focus on the development of their social and economic infrastructures. Otherwise, they may consume all their energies and resources to mitigate these issues and may indulge in an unending war. Keeping these issues aside, if the countries will make a sincere effort in true letters and spirits, to achieve sustainable economic development in the light of UN Agenda 2030, then a drastic global transformation may be observed. Kılkış (32) has made a comprehensive analysis of the SOIS in Brazil, India, China, Russia, Turkey, and Singapore and has developed the Sustainable Innovation Index (SII) to check the performance of their respective economies. In the research study, he used four layers of SOIS; system analysis, knowledge production, technological innovation, and system efficiency. In the system analysis, support mechanisms and functional dynamics are included. Similarly, knowledge production can be observed through paper analysis, which means how many papers have been produced based on novel ideas. The third layer consists of patent analysis which means how many patents have been granted to local companies which reflect technological innovation. The fourth layer provides a comparative analysis to check the system’s efficiency. As well as developing countries are concerned, they have basic institutional flaws, a lack of implementation of rules and regulations, and deficiencies in constitutional implementations. For them, it would be objective and initially, they must promote basic infrastructures to support the innovation activities at individual and collective levels.

Furthermore, Altenburg and Pegels (33) have conducted a comprehensive analysis of SOIS through green transformation. According to the authors, all the developments in advanced countries have been held at the cost of environmental destructions and natural resource depletions which have created environmental problems like global warming, and it has threatened human livelihood. As well as developing countries are concerned, they are exporting agricultural products, primary goods, and other natural raw materials. There is high pressure on the government due to scarcity of food, hyperinflation, increasing poverty, and deficiency of the provision of utilities and other social facilities. To control this issue, there is an immense need to introduce SOIS to control further deterioration in a green environment. The authors have further emphasized improvement in evolutionary innovation systems, which is impossible without better and committed governance. The economies must develop a policy framework that gradually addresses the remedial measures for methodological issues for short-term, medium-term, and long-term objectives. The starting point of this development is to introduce environmentally sustainable technologies which is the need of the day.

The SOIS is considered a new approach to innovation systems. In this system, the prime objective is to generate innovations that reduce environmental pressure. In general, there are certain innovation systems like global Innovation Systems, Regional Innovation Systems, and National Innovation Systems. In these systems, innovation is generated without catering to the sustainability aspects (34). SOIS is used as a new tactic for innovation systems to fill this research gap. The environmentally conducive technologies can be generated with the cooperation’s global stakeholders. To develop an effective SOIS, technological knowledge plays a significant role. To develop technological expertise, the commitment of the government is necessary for the shape of policy formulations and regulations with the protection of local industries. Corsi et al. (35) have suggested that developing countries enhance technological information through globalization and technology transfers from developed to developing countries. Technology cooperation among developing and developed economies is another mode to relocate knowledge-intensive activities. Similarly, developing countries may learn from the Organizations for Economic Cooperation and Development (OECD) countries about innovation policies, technological knowledge, and implementation criteria to promote economic development in their respective economies.

### 2.3. The mechanism for implementing sustainability oriented innovation system

It is a general presumption that innovative firms are more productive and efficient as compared to non-innovative firms. For instance, Camisón-Haba et al. (36) have investigated technology-based economic development and explained that innovative firms are more contributive to economic development than non-innovative firms. Other studies like Ahmad et al. (37) and Silvestre and Ţîrcă (38) have emphasized the promotion of innovative activities because it is considered one of the key drivers of sustainability and amplifies economic development in respective countries. Economic development is the composition of economic growth and social sector development. It reflects the progress in economic as well as social indicators of the country. Innovation can be applied in all sectors of the economy. For instance, innovation in renewable energy, innovation in product diversification, and innovation in processes help in achieving economic development in the country. Moreover, Tabrizian (39) has underlined the importance of innovation in the renewable energy sector to speed up the pace of economic development in developing countries. According to the study, those economies which are reinforcing renewable infrastructures may get competitive advantages in global markets. It is admitted fact that developing countries are facing serious shortfalls in the energy sector to meet the respective demands. Improper planning of energy resources and disproportionate surge in population growth have imbalanced the general equilibrium of developing economies. To fulfill the need for the industrial sector as well as for household consumption of energy, it is indispensable to make innovations in the energy sector. The main rationale behind this is that natural resources are depleting day by day and there is an immense need to move towards alternative energy resources to save a green environment in developing countries.

Zartha Sossa et al. (40) have investigated the effects of an innovation system for attaining sustainability in the Colombian region. Their study indicated that there is a lack of disarticulation between the policies related to sustainability in the innovation system which may amplify economic development. Further suggested that socio-environmental considerations should be included in the innovation system and to amplify the economic growth in the country, there is an immense need to channel natural resources for optimum production.

Uğurluay and Kirikkaleli (41) have investigated that Innovation Systems, in high-income countries, play a significant and positive role in developing cutting-edge technologies which ultimately result in promoting economic activities. Developing countries must focus on technology transfer from developed economies to attain better economic growth. To implement SOIS in developing countries, certain prerequisites are essential to be achieved.

To implement the SOIS to foster economic stability in developing countries, there are certain prerequisites. First and foremost, the thing is respective governments may develop basic economic and social infrastructure and do sincere efforts for the transformation of their respective economies. Keeping all political and sectarian disputes aside, political leaders should join hands for the prosperity and development of their nations. Innovation is an ongoing process and SOIS reflects that innovation which is decoupling economic growth from environmental pressures (40). Silvestre and Ţîrcă (38) have concentrated on the promotion of innovation due to its role in transforming individuals, societies, organizations, and communities. It also requires immediate actions by governments and industries to adopt environmentally friendly innovation systems or eco innovative technologies to foster sustainable development. The authors have suggested three main areas of change before the implementation of SOIS; technology, management, and policy. The respective governments in developing countries must focus on basic infrastructure for technology and innovation development as a foundation for implementing SOIS. Secondly, the management of capital and natural and human resources is one of the key factors which may assist in implementing SOIS. The provision of industrial, labor, and investment policies are a third key factor for implementing the SOIS to increase economic stability. Another key sector for developing countries is the external sector, through which they can progress their economic strength and promote innovative activities. External sector development is one of the key factors for sustainable economic strength. The Exports Led Growth (ELG) hypothesis has proven the progress of transitional economies. If the exportable items are based on semi-manufactured or manufactured goods, then the external sector would develop at a greater pace (42). In this regard, Ahmed and Mahmud (43) have conducted a study related to the determinants of innovation in the manufacturing sector in Pakistan. The authors have highlighted the significance of innovation in achieving competitiveness and comparative advantages which ultimately help in getting high economic growth. Further, that innovation plays a vital role in getting the economic stability of Pakistan. It indicated the significance of innovative and automated systems which may help achieve the economic stability of developing countries effectively. There are multiple factors in introducing innovation in manufacturing sectors like firm size, the scale of production, and type of product; less elastic or more elastic, final goods, or semi-manufacturing goods. Similarly, Aldieri and Vinci (44) have studied the relationship between firm size and sustainable innovation in large international firms. Data were collected from firms in Japan, the United States, and Europe and explained the positive connection between firm size, technological spillovers, research and development activities, and sustainable innovation.

## 3. Methods and data

To see the impact of SOIS which consists of the innovation input index and innovation output index on economic stability in developing and developed countries, panel data are used. The period of analysis consists of 2011 to 2021. Certain parameters like GDP growth, innovation input index, and innovation output index have been used as a proxy for economic stability and SOIS, respectively. The innovation input index consists of five main subheads: Institutions index, human capital, and research index, infrastructure index, market sophistication index, and business sophistication index. The output innovation index consists of two subheads: the knowledge and technology output index and the index of the creative output. The data was collected from different international sources. Macroeconomic parameters like the GDP Growth Rate of respective countries have been collected from World Development Indicators (WDI) a publication by the World Bank and Key Indicators of Asia and Pacific 2021, a publication by the Asian Development Bank.

The information about the innovation input index and innovation output index for the respective countries are collected from the Global Innovation Index (GII) which is an annual publication of WIPO (World Intellectual Property Organization). The data was collected for the most innovative countries, segmented based on WIPO (World Intellectual Property Organization). Those countries belong to High-Income Countries (Switzerland, Sweden, and the United States of America), Upper Middle-Income Countries (China, Bulgaria, and Malaysia), Lower Middle Income (Vietnam, India, and Ukraine), and from Low-Income Countries (Rwanda, Tajikistan, and Malawi) are included in the analysis for the period of 2011 to 2021.

### 3.1. Theoretical foundation of the variables

It is a fundamental truth that innovation has a positive and significant effect on economic growth which is supported by Schumpeter’s innovation theory (1934) (45). Schumpeter has claimed that development is deemed a historical process of structural changes. These changes are driven by innovative ideas and their execution in the tangible form either in the shape of products, services, or industries. Schumpeter has advised the nations to adopt novel and innovative methods of production to spur their economic growth. Innovation may assist in the opening of new market avenues at domestic and international levels and develop a competitive business environment along with industrialization which may revolutionize the economic systems of the country.

### 3.2. Empirical strategy

To check the role of SOIS in the economic stability of respective countries, an econometric model has been developed. SOIS has been translated through the innovation Input Index and innovation output index. To estimate the regression parameters, the software Stata 16 SE has been used by developing a set of panel data. The Fixed Effect Method (FEM) was used to ascertain the empirical results. The detail of the formulation of regression equations as explained below. There are two regression equations, in which the relationship between outcome and explanatory variables has analyzed. Regression Eq. (1) is explaining the effect of the innovation input index on the economic stability of respective countries. Eq. (2) has been formulated to see the impact of the innovation output index on economic stability in the respective countries.

In the light of the regression model, the effects of the innovation input index on economic stability sets out the following form:


(1) $$\begin{array}{l} GDPG_{it}=\alpha +\beta_{1}INS_{it}+\beta_{2}HCR_{it}+\beta_{3}INFR_{it}+\\ \beta_{4}MRSOP_{it}+\beta_{5}BSOP_{it}+\varepsilon_{it} \end{array}$$

where:

GDPG is the growth rate of gross domestic product, INS is institutions index, HCR is human capital and research index, INFR is the infrastructure index, MRSOP is the market sophistication index, BSOP is business sophistication index, ε is the error term or residual term, and *i* = 1, 2, …, 12 while *t* = 1, 2, …, 11.

In this model, GDPG is the outcome variable that measures economic stability in respective countries. Institutions Index (INS), Human Capital and Research Index (HCR), infrastructure index (INFR), Market Sophistication index (MRSOP), and Business Sophistication Index (BSOP) are explanatory variables. The institution index consists of the political environment, regulatory environment, and business environment of the respective countries. Similarly, Human Capital and Research Index is consisting of three components: education, tertiary education, and research and development situation in respective countries. The infrastructure consists of three components Information and communication technologies (ICTs), general infrastructure, and ecological sustainability. The market Sophistication index (MRSOP) is calculated based on Credit Investment, Trade diversification, and market scale in the respective countries. The business Sophistication Index (BSOP) is based on Knowledge workers, Innovation linkages, and Knowledge absorption. In other words, how many people are engaged in imparting the latest knowledge to society for the betterment of the business environment? There is a significant impact of innovation linkages among the business entities on the economic stability of the country. If these linkages are strong and persistent then there is the possibility of positive and smooth growth in the country.

Therefore, to study the effect of the innovation output index on the economic stability of the most innovative countries sets out the following shape:


(2) $$GDPG_{it}=\chi_{0}+\chi_{1}KNTO_{it}+\chi_{2}CO_{it}+\mu_{it}$$

where:

GDPG is the GDP growth rate, KNTO is knowledge and technology output, and CO is creative output.

Eq. (2) explains that the GDP Growth rate is a dependent variable while the knowledge and technology outputs index (KNTO) and Creative Outputs index (CO) are independent variables. It is presumed that these two independent variables have a positive effect on the economic stability of the countries. The knowledge and technology output index (KNTO) are consisting of three components: Knowledge creation, Knowledge impact, and Knowledge diffusion. The Creative Outputs index (CO) has been developed based on three components: intangible assets, creative goods and services, and online creativity (Global Innovation Index 2021, WIPO).

## 4. Empirical results and discussions

To test the stationarity of the data, the panel unit root test (Levin, Lin and Chu Test) has been applied. This test is significant which indicates that mean, variance and covariances are not changing over time. It has been observed that except CO all the variables are stationary at first difference. The detail of the stationarity test results has been mentioned in Table 1.

**Table 1:** Results of stationarity test (Levin, Lin and Chu Test).

Variables	LLC at level	LLC at first difference
D(BSOP)	4.60 (1.00)	−10.85 (0.00)^*^
D(GDPG)	−2.27 (0.15)	−17.45 (0.00)
D(INFRA)	8.31 (1.00)	−35.71 (0.00)
D(INST)	3.54 (0.99)	−44.54 (0.00)
D(HCR)	0.35 (0.63)	−5.45 (0.00)
D(KTO)	6.34 (1.00)	−40.93 (0.00)
D(CO)	−3.65 (0.00)	−25.61 (0.00)

The values in parenthesis are probability values which should be less than 0.05.

To estimate Eqs. (1)–(2), Fixed Effect Methods (FEM) have been used due to the nature of the data and the results of the Hausman test, as mentioned in Table 2.

**Table 2:** Results of the Hausman test (innovation input index).

Test summary	Chi-Sq. Stat	Chi-Sq. d.f.	*p*-value
Cross-section random	28.36	5	0.000

Table 2 represents the results of the Hausman test which indicate that the Chi-Square Stat is significant with a Prob Value less than 0.05. Due to its significance, the use of a fixed effect estimator is appropriate for ascertaining empirical results (46). Table 3 explains the effects of the innovation input index on economic stability, respectively.

**Table 3:** Effect of innovation input index on economic stability dependent variable: LOG (GDPG) method: panel EGLS (cross-section weights).

Variable	Coefficient	*t*-statistic	Prob.
D(INST)	0.016	1.392	0.167
D(INFRA)	0.026	2.701	0.008
HCR	−0.002	−0.108	0.914
BSOP	0.007	1.481	0.142
MRTSOP	0.031	3.693	0.001
C	0.876	1.521	0.132
*R*-squared	0.811	Durbin–Watson stat	1.719
*F*-statistic	26.502	Prob(*F*-statistic)	0.000

HCR, human capital and research index; INFRA, infrastructure index; INS, institutions index; MRTSOP, market sophistication index; BSOP, business sophistication index.

Table 3 explains the empirical results of the effects of the Innovation Input Index on the economic stability of the respective countries. According to the results, the infrastructure index has a significant and positive effect on the economic stability of the countries. In other words, Infrastructural improvement plays a pivotal role in the development of a country. The T. Stat value (2.70) indicates the significant and positive effects of INFRA on the economic stability of the countries. The infrastructure of a country, which consists of roads, dams, utilities like electricity, Gas and Water resources, transportation, and communication, provides momentum for the development of a country’s economic and social structure. The pace of development amplifies with better infrastructure. The results of the present study also emphasize the importance of the infrastructure of respective countries. These results are consistent with prior studies like (47, 48).

The present study indicates a positive and significant effect of market sophistication, which consists of three main pillars: credit investment, trade diversification, and market scale on the economic stability of the most innovative countries. The T. Stat (3.693) indicates a positive and highly significant effect of market sophistication on economic stability. All three pillars of market sophistication are very important for generating economic activities in the country. For instance, trade diversification creates multiple opportunities for enhancing the trade volume as well as foreign remittances.

Similarly, credit investment is another important tool for promoting economic and business activities in the country. The results of the present study emphasize the significance of market sophistication in increasing the economic strength of respective countries. The result of this study is explaining the importance of business sophistication. The world is lacking behind in this field. The T. Stat (1.481) indicates an insignificant effect on the economic stability of the respective countries. Business sophistication consists of knowledge workers, innovation linkages, and knowledge absorption. There is huge potential in all respective economies if they develop knowledge workers and innovation linkages among developing and developed economies. There is an immense need to develop such a comprehensive and integrated mechanism for innovation linkages among the countries. The results further explain the overall significance of the regression model which is reflected by *F*-Stat. The probability value should be less than 0.05 for overall model significance. The *R*-Square value determines the goodness of fit of the model. *R*-Square value ranges from 0 to 1. If the value of *R*-Square is closer to 1, it means that regression model is explaining the observed data in a good manner. In the present study, value of *R*-Square is 0.81 which represents goodness of fit of the model. The D.W value explains the presence of autocorrelation. If the value of D.W is close to 2, it means that there is no autocorrelation. In the present study, the D.W Stat is 1.70 which is closer to 2 and reflects no autocorrelation in the model.

Above Table 4 represents the results of the Hausman test which indicate that Chi Square Stat is significant with Prob Value (0.025).

**Table 4:** Results of the Hausman test (innovation output index)

Test summary	Chi-Sq. Stat	Chi-Sq. d.f.	*p*-value
Cross-section random	7.37	2	0.025

Table 5 explains the effects of the innovation output index on the economic stability of the respective countries. Table 5 highlights the positive and significant effect of the knowledge and technology output index (KNTO) on economic stability. The T-Stat (4.83) indicates that KNTO has a highly significant effect on the economic stability of the countries. This index is consisting of three components: knowledge creation, knowledge impact, and knowledge diffusion. These results are consistent with the findings of prior studies of Kaneva and Untura (49) and Mao et al. (50). Similarly, CO (Creative Outputs) has a merely significant effect on the economic stability of the countries. These results are consistent with prior studies of Bennett and Nikolaev (51) and Hawkins (52). These studies have concentrated on the significance of innovation for the progress and growth of economic activities in developed as well as developing countries.

**Table 5:** Effect of innovation output index on economic stability dependent variable: GDPG method: panel EGLS (cross-section weights).

Variable	Coefficient	*t*-statistic	Prob.	
KNTO	0.028	4.836	0.000	
CO	0.006	1.059	0.292	
C	0.169	0.433	0.665	
*R*-squared	0.816	Durbin–Watson stat		1.772
*F*-statistic	36.621	Prob(*F*-statistic)		0.000

KNTO, knowledge and technology output; CO, creative output.

## 5. Conclusion and limitations

The present study is an attempt to review the effects of SOIS on the economic stability of the most innovative countries. To see the impact of SOIS on economic stability, the innovation Input Index and Innovation Output Index have been used as a proxy for SOIS. Economic Stability is measured through the annual growth rate in their respective GDPs. A set of panel data have been developed based on several cross-sections (12 Countries). The Fixed Effect Method (FEM) has been used to ascertain empirical results. The main results are in favor of positive and significant effects of components of the innovation input index (Human Capital & Research and Infrastructure) on the economic stability of respective countries. Similarly, the Innovation Output Index has a positive and significant impact on the economic stability of respective countries.

Due to Novelcoronavirus-19, in the global economy, developed as well as developing countries have shown a drastic decline in their social and economic sector’s growth. The manufacturing and services sectors have taken adverse effects in the developing economies due to COVID-19. It is not certain when this pandemic will end but the economies must prioritize their preferences in policy formulation and their abrupt implementation keeping in view the ease of the public and increasing economic strength of their respective countries. It is very painful to see the miseries of the deprived people and the failure of health systems, especially in India during COVID-19. The government must refrain from blame games and sincerely take effective measures to provide basic health provisions to the public. These priorities have already been discussed under the UN Agenda 2030 for sustainable development around the globe. These Agenda items are reflecting the transformational objectives of member countries. Keeping all political, social, and economic conflicts aside, all the economies must join their hands for the prosperity of their respective nations under the Charter of the UN. The UN must play its pivotal role in resolving the deadlocks among the nations like the matter of Palestine, Kashmir, and other disputes through dialogues by using their forum. Those resources should be used for the betterment of their respective inhabitants.

To implement SOIS in developing countries, certain prerequisites are essential to be achieved. These countries must focus on technology transfer from developed economies to developing countries to attain better economic growth. The respective governments in developing countries may focus on the provision of basic infrastructure for technology and innovation development as an underpinning source for implementing SOIS. Managing natural, human, and capital resources is one of the key factors that may assist in implementing SOIS. The provision of conducive industrial and investment policies is key to implementing SOIS.

It is generally impossible to capture the issue from all aspects so there are certain limitations of the study. First, as per the declaration of the Global Innovation Index report, 2019–2020, the 12 most innovative countries are included in the analysis. To take a more comprehensive picture of the study, all 132 countries can be included. Second, this study used secondary data; however future studies can use primary data for analysis. Third, future studies may add other variables for analysis. Additionally, Future studies may focus on the effects of SOIS on economic stability in regional blocks like the EU, ASEAN, G-20, and OECD.

## Data availability statement

The raw data supporting the conclusions of this article will be made available by the authors, without undue reservation.

## Author contributions

FM initiated the basic idea, wrote the main part of the manuscript, and built the article structure. LW reviewed and improved the manuscript. QS and MS contributed to the methodology of this study. All authors contributed to the article and approved the submitted version.

## Funding

This study was supported by the National Social Science Foundation of China [22&ZD081].

## Conflict of interest

The authors declare that the research was conducted in the absence of any commercial or financial relationships that could be construed as a potential conflict of interest.

## Publisher’s note

All claims expressed in this article are solely those of the authors and do not necessarily represent those of their affiliated organizations, or those of the publisher, the editors and the reviewers. Any product that may be evaluated in this article, or claim that may be made by its manufacturer, is not guaranteed or endorsed by the publisher.
